# 

**Published:** 1891-10

**Authors:** 


### BOOKS AND PAMPHLETS RECEIVED.

Bulletin de la Société Zoölogique de France, Paris.

Der Zoologische Garten, edited by *Dr. F. C. Noll*, Frankfort, a. M.

Annalen des K. K. Naturhistorischen Hofmuseums, redigirt von *Dr. Franz Ritter*, von Hauer Wein 1891. Bd. vi. No. 1-2.

Jahresbericht der Naturforschenden Gesellschaft ln Emden- 1889-90.

Verslag van den Westand van het K. Zoo.-Bot. Genoolschap. Te S. Gravenhage. 1890.

Journal Bombay Natural History Society. Vol. vi, No. 1.

